# Utilizing heart rate variability to predict ICU patient outcome in traumatic brain injury

**DOI:** 10.1186/s12859-020-03814-w

**Published:** 2020-12-14

**Authors:** Ping Zhang, Tegan Roberts, Brent Richards, Luke J. Haseler

**Affiliations:** 1 Menzies Health Institute QLD, Griffith University, Gold Coast, Australia; 2 School of Medical Science, Griffith University, Gold Coast, Australia; 3 School of Medicine, Griffith University, Gold Coast, Australia; 4 Gold Coast University Hospital, Gold Coast, Australia; 5 School of Physiotherapy and Exercise Science, Curtin University, Perth, Australia

**Keywords:** ECG, Time series, HRV, Feature extraction, Euclidean distance, Patient outcome, ICU

## Abstract

**Background:**

Prediction of patient outcome in medical intensive care units (ICU) may help for development and investigation of early interventional strategies. Several ICU scoring systems have been developed and are used to predict clinical outcome of ICU patients. These scores are calculated from clinical physiological and biochemical characteristics of patients. Heart rate variability (HRV) is a correlate of cardiac autonomic regulation and has been evident as a marker of poor clinical prognosis. HRV can be measured from the electrocardiogram non-invasively and monitored in real time. HRV has been identified as a promising ‘electronic biomarker’ of disease severity. Traumatic brain injury (TBI) is a subset of critically ill patients admitted to ICU, with significant morbidity and mortality, and often difficult to predict outcomes. Changes of HRV for brain injured patients have been reported in several studies. This study aimed to utilize the continuous HRV collection from admission across the first 24 h in the ICU in severe TBI patients to develop a patient outcome prediction system.

**Results:**

A feature extraction strategy was applied to measure the HRV fluctuation during time. A prediction model was developed based on HRV measures with a genetic algorithm for feature selection. The result (AUC: 0.77) was compared with earlier reported scoring systems (highest AUC: 0.76), encouraging further development and practical application.

**Conclusions:**

The prediction models built with different feature sets indicated that HRV based parameters may help predict brain injury patient outcome better than the previously adopted illness severity scores.

## Background

Traumatic brain injury (TBI) is increasingly considered to be an important global health priority as it results in a large number of deaths and impairments leading to permanent disabilities [1, 2]. TBI patients are almost always admitted to an intensive care unit (ICU) and receive high level life support and continuous monitoring. Prediction of clinical outcome in these patients, based on the continuous monitoring of physiological signals, may allow for early identification of injury severity and ultimately guide interventional strategies which may improve survivability rates, or in cases of poor outcome, inform end-of-life decisions. Currently, there are several ICU scoring systems in place to measure the severity of TBI including the Acute Physiology and Chronic Health Evaluation (APACHE II and the updated versions APACHE III/IV) [3–6], Simplified Acute Physiology Score (SAPS II) [7], Multiple Organ Dysfunction Score (MODS) [8], the Sequential Organ Failure Assessment (SOFA) [9], and Injury Severity Score (ISS) [10, 11], with a comprehensive review of ICU scoring systems by Rapsang and Shyam [12]. These scores correspond to risk of death, and are commonly used to predict TBI patient outcomes. The scores are calculated based on patient characteristics, including age, chronic health status, major medical and surgical disease categories, acute physiologic abnormalities, pre-existing functional limitations, major comorbidities and ICU admission variables (with a slight difference of variables used in each scoring system). Strong correlation between these scores may exist [12, 13]. The predictive ability of these scoring systems on ICU patient outcome has been evaluated previously [14, 15], and the APACHE and SAPS scoring systems have been prospectively verified [16, 17]. Recent investigations attempted to expand APACHE III with additionally available clinical records, however only the potential to improve was reported [18]. None-the-less, these scores do not take into account the heterogeneity that exists between patients due to the discrepancies in initial TBI presentations and the evolution of secondary brain injurie.

### Heart rate variability and traumatic brain injury

The Electrocardiogram (ECG) is a non-invasive measure of the heart’s overall electrical activity and is measured continuously during a patient’s stay in the ICU. ECG waveform interpretation has provided the basis for clinical diagnosis of progressive heart disease and lethal arrhythmias. A novel extension of ECG monitoring is assessing the beat-to-beat variation in heart rate (HR) termed Heart Rate Variability (HRV). In a healthy individual, autonomic nervous system (ANS) activity is a key regulator of HR; changes in parasympathetic and sympathetic nervous system during normal circadian rhythm lead to HR fluctuations. HRV is a correlate of cardiac autonomic regulation and has been identified as a promising ‘electronic biomarker’ of disease severity and predicting patient outcomes. The Chinese physician, Wang Shu-he (265–317 A.D), noted the variability of the heart as an indicator of the critically ill: “If the pattern of the heart beat becomes as regular as the tapping of a woodpecker or the dripping of rain from the roof, the patient will be dead in 4 days” [19].

HRV is the variation in time between consecutive heart beats (RR interval) and can be analysed in both time and frequency domains. Time domain analysis calculates and assess the overall RR interval time series and frequency domain analysis quantifies the overall variability as frequency of ANS function.

**Fig. 1:**
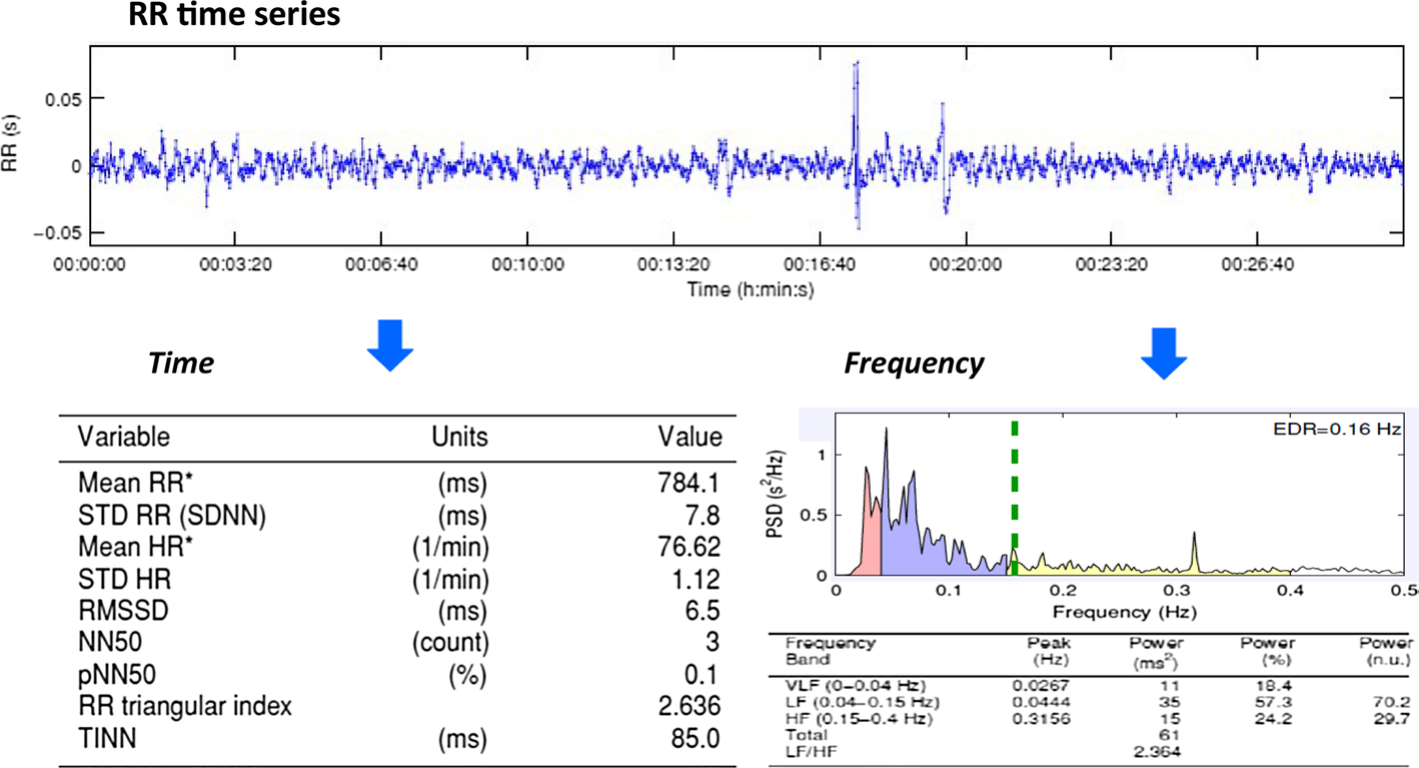
Example of RR time series analysis in the Time and Frequency domains. The RR time series are derived into the time and frequency domains. Time domain calculates overall variability within the sample, and frequency domain calculates autonomic modulation. (Kubios software, version 2.2, Biosignal Medical Group, Kupio, Finland)

HRV analysis in time domain is the simplest quantification method of HRV, calculated on a beat-to-beat basis. In the literature, RR intervals of normal sinus rhythm are denoted as normal-to-normal (NN) beats. Standard deviation of the NN interval (SDNN) reflects all the cyclic components responsible for variability within the recording time period, for example a 24-h period. The square root of the mean square differences of successive NN intervals (RMSSD) reflects high frequency variations in heart rate. RMSSD is highly correlated with both NN50, the number of successive NN beats that differ by more than 50 ms and pNN50 the percentage of NN50 over the entire NN series. The statistical properties of RMSDD are preferred to pNN50 and NN50 (Fig. 1). Whilst time domain methods can be calculated from short 5 min signals to entire 24 h signals, it is recommended that comparisons between different recording lengths in the time domain be avoided as HRV is not a stationary process.

Frequency domain measurements estimate the distribution of absolute or relative signal energy into four frequency bands. Frequency Domain Analysis shows how much of a signal lies within one or more frequency bands (ranges). The Task Force of the European Society of Cardiology and the North American Society of Pacing and Electrophysiology [20] divided HR oscillations into ultra-low-frequency (ULF), very-low-frequency (VLF), low-frequency (LF), and high-frequency (HF) bands. The power within these frequency ranges represents the overall variance, expressed as milliseconds squared (ms^2^), implying the greater the power, the greater variation (Fig. 1). Total Power (TP) is a measure of overall variance in RR intervals accounting for all sources, nervous, hormonal and circadian. It is a measure of overall variation in HR—the greater the TP the more variance there is within the time series, therefore the heart can adapt quicker to stimulus. LF and HF may also be measured as normalised (nu) (LFnu, HFnu) representing the relative value of each power whilst correcting for TP. This allows comparison between individuals as it accounts for their individual variance. Additional details about HRV measures in both time and frequency domains can be found in reference [21].

Traumatic brain injury (TBI) is a sub-category of patients admitted to ICU for critical care. The latest annual incidence of TBI worldwide indicated that incidence is currently 295/100,000 for all ages [22]. By 2030, brain injuries due to traffic accidentals are expected to rise to the 7th leading cause of death [23]. Treatment of TBI is confounded by the wide heterogeneity between patient presentations, extensive comorbidities and the widespread secondary complications that evolve from the primary damage. This diversity between patients makes injury severity difficult to gauge, thus clinicians are always looking for newer, patient specific indicators of secondary brain injury evolution and outcomes. A method of predicting patient outcome may assist in clinical decisions and allow for more informed discussions in family meetings. Thus, a model of patient outcome would be a useful tool if incorporated into the ICU workflow.

Autonomic impairment after acute TBI has been associated independently with increased morbidity and mortality [24, 25]. HRV, as a correlate of ANS regulation of HR, provides an ideal physiological marker to form the basis of a prediction model. Changes in HRV for brain injured patients have been reported in several studies [26–30]. In these studies, both time and frequency domains of ECG signals were analysed. Winchell et al. [31] studied the effect of alterations in HRV on mortality in a surgical ICU population, and reported that low TP (reduced autonomic tone) and high HF/LF ratio (relative lack of sympathetic tone) were associated with increased mortality. A low HF/LF ratio (relatively high sympathetic tone) was also found to be associated with increased survival, especially in patients with low autonomic tone. Sykora et al. [26] reported that over long term-indiscriminate averaging, autonomic impairment was associated with increased HF powers and decreased LH/HF ratio, as measured by HRV. This was significantly associated with increased mortality after TBI, independent of intracranial pressure and cerebral perfusion pressure. For every increase in relative HR power, the odds for mortality increased by 4.6%. Haji-Michael et al. [32] showed that brain injured patients had reduced HRV, such as a lowered total power variability of RR and a lowered LF/HF ratio, whereas recovery of HRV was associated with an improved outcome. Kox et al. [33] also investigated the association of HRV and the innate immune system response in brain injured patients. They found that higher levels of HFnu were correlated with attenuated levels of plasma Tumour Necrosis Factor Alpha (TNF-α), indicating a reduction in inflammatory mediators and thus demonstrating an immune-suppressive mechanism of action. In the subgroup of patients with intracranial haemorrhage, increased intracranial pressure was correlated to an even higher degree of HFnu and immune suppression. Association of brain death with HRV responses was reported in the study of Baillard et al. [34], and Piantino et al. [35] also reported that children who progressed to brain death exhibited lower HRV in both time and frequency domains. These findings suggest ANS dysfunction may be implicated with poor outcomes and indicate that HRV may be a promising predictor of adverse outcomes in TBI patients.

The findings of the above studies were based on univariate analysis. In addition, most research regarding TBI and HRV was only carried out with periodic calculations (5 min or 10 min recordings), within the acute phase of brain injury (72 h post ictus) and free of interventions and confounding medication. This study aimed to investigate the aspects of continuous HRV collection from admission across the first 24 h of stay in the ICU in severe TBI patients and utilize the continuous HRV measurement to develop a patient outcome prediction system. The advantages of using HRV analysis is that it utilises cardiovascular bio-signals that are readily available, pre-existing standards of care, patient specific and inexpensive, which means that earlier identification of outcome in these patients may be improved without an increase in cost of care.

## Methods

**Fig. 2:**
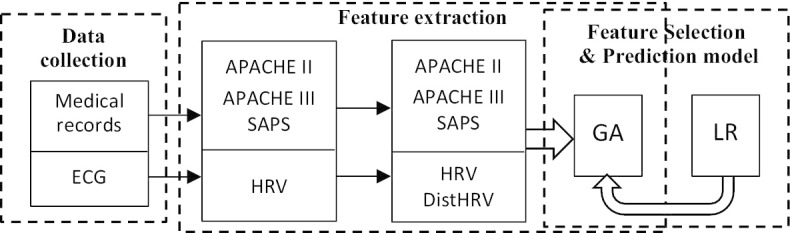
A diagram of the proposed method. APACHE II, APACHE III and SAPS scores were calculated based on medical records. HRV parameters were calculated based on the patient ECG data, and these parameters and the distribution of each of the HRV parameters across each of 8 continuous time points were used as the input variables (features) to the classification model. The classification model used here is logistic regression. A genetic algorithm (GA) was used for feature selection to find variable combinations that build the most accurate prediction model.

The goal of this study was to utilize the 24 h continuous HRV measurement and develop a surviving prediction system applied to ICU TBI patients. For this study, the model development can be described as 3 parts: data collection, feature extraction and building of prediction models. See Fig. 2.

### Data collection

Electrocardiogram (ECG), mechanical ventilation parameters, medication and the Glasgow Coma Scale (GCS) are standards of care that are continuously monitored throughout a patient’s Intensive Care Unit (ICU) stay. For this study, 26 ICU patients with diagnosed TBI were sampled [36]. Medical records and 24 h ECG data were collected from the patient bedside GE monitor via a separate output at 300 Hz. Twenty-one of these patients survived ICU admission to be discharged to the ward without complications (survivors); while five patients did not survive ICU (non-survivors). Age, gender, etiology of injury and diagnosis were also recorded upon admission to the ICU and inclusion to the study.

### Feature extraction

Based on the patient medical records, APACHE II [4], APACHE III [5], and SAPS II [7] scores were calculated for each patient. The 24-h ECG signals were analysed using Kubios HRV software [37] (version 2.2), and HRV parameters were calculated over consecutive 30-min epochs in both the time and frequency domains. The parameters calculated based on time domain included: SDNN, RMSSD and CVRR (the coefficient of variation of R-R intervals). Frequency analysis included LF, HF and LF/HF ratio, representing sympathetic, parasympathetic and sympathovagal balance respectively. Normalised units, LFnu and HFnu were calculated taking into account TP.

To utilize the consecutive HRV parameters calculated on both time domain and frequency domain, Euclidean distance between a time series and a uniformed distribution was used to measure the variance of the calculated HRV parameters during the consecutive time periods. For example, for a n consecutive 30-min ECG signal, there are a number of n SDNN calculated. To measure the fluctuation of SDNN across the n consecutive epochs, a Euclidean distance between the vector of n values of SDNN and a baseline with a vector of n zeros was calculated as a feature of the corresponding time point of the patient. For patient i, a Euclidean distance feature based on a n-consecutive 30-min ECG segments, DistFi_nseg, can be calculated with the formula below:

$${\text{DistFi}}\_{\text{nseg}} = \sqrt {F_{i1}^{2} + F_{i2}^{2} + \cdots F_{in}^{2} } \quad i = 1,2, \ldots M$$

where *M* is total number of the patients, $F_{i1} \ldots F_{in}$ are the n features to be calculated, for example HR, RR, SDNN, LF_Hz etc. For this study, a total number of 20 HRV parameters calculated from Kubios were extracted (Table 1) and 20 Euclidean distance features based on these parameters were calculated for each patient.

**Table 1:** List of HRV parameters/features extracted from ECG signals

HRV parameter	Description (30 min ECG)
HR	The mean heart rate
RR	The mean of RR intervals
SDNN	Standard deviation of RR intervals
RMSSD	Square root of the mean squared differences between successive RR intervals
CVRR	Coefficient of variance of RR intervals
VLF_Hz	Peak frequency for VLF band
LF_Hz	Peak frequency for LF band
HF_Hz	Peak frequency for HF band
VLF_ms_sq	Absolute power of VLF band
LF_ms_sq	Absolute power of LF band
HF_ms_sq	Absolute power of HF band
VLF_perc	Relative powers of VLF band
LF_perc	Relative powers of LF band
HF_perc	Relative powers of HF band
LF_nu	Power of LF band in normalized unit
HF_nu	Power of HF band in normalized unit
Total_ms_sq	Total spectral power
LF/HF	Ratio between LF and HF band powers
SD1	Standard deviation of Poincaré plot, nonlinear method to measure short-term variability
SD2	Standard deviation of Poincaré plot, nonlinear method to measure long-term variability

Due to the nature of the data we collected, which included 2 patients that did not survive for more than 12 h from admission, for this study a length of 8 consecutive time points were used for calculating the Euclidean distance features (DistF). For each patient at each time point (a 30 min time period) the corresponding DistF was calculated based on the following 8 time points (inclusive).

For example, for a patient with 24 h ECG data collected there would be HRV data collected at 48 time points during each 30 min. Therefore, there will be 41 DistF calculated corresponding to 41 time points, which can be used for building the prediction model. The data structure for this patient would be like that shown in Table 2. Prediction models will be built with the sets of selected features from the whole set.

**Table 2:** Data structure for one patient

Time point (Time period)	HRV features				HRV Euclidean distance features				
	HR	RR	……	SD2	Time points for calculation	DistHR	DistRR	…	DistSD2
1[0,0.5)*	57.7	1040.7	……	8.6	1–8	166.9	2884.1	32.6	166.9
2[0.5,1)	56.3	1066.0	……	12.4	2–9	167.3	2876.5	33.4	167.3
3[1,1.5)*	58.2	1030.6		10.0	3–10	168.6	2852.6	33.1	168.6
4[1.5,2)	59.4	1014.3		12.2	4–11	169.0	2846.2	33.3	169.0
5[2,2.5)*	60.2	998.8		11.3	5–12	168.4	2856.6	33.2	168.4
6[2.5,3)	58.2	1032.0		10.8	6–13	167.1	2880.3	33.1	167.1
7[3,3.5)*	60.4	996.3		14.8	7–14	167.1	2879.9	33.2	167.1
8[3.5,4)	61.5	975.8		11.4	8–15	165.9	2900.1	32.2	165.9
⋮	⋮	⋮	……	⋮	⋮	⋮	⋮	…	⋮
40 [19.5,20)*	62.8	957.8	……	15.1	40–47	259.3	2019.8	…	40.2
41 [20,20.5)	68.5	877.1	……	12.0	41–48	272.3	1870.5	…	39.0

^*^[0, 0.5) represent the first 30 min (half an hour) of the ECG record.[20, 20.5) represents the ECG record from hour 20–20.5 h time point

### Prediction models

Logistic regression (LR) is consistently used in the literature for the transformation of ICU severity scores into a probability of patient death in hospitals [12, 17, 18]. To compare the predictability of HRV parameters with that of APACHE scores, LR was used as a classification method in this research. To search the best set of HRV features for predicting the probability of individual mortality, a genetic algorithm (GA) was applied for this study.

A GA is a search heuristic to find optimal solutions for a problem. In this study, it was used for selecting the best feature set for a classification/prediction model, which was built with the LR method. The best feature sets were defined as the ones that discriminate the best between survivors and non-survivors. The discrimination can be measured by false positive rate and false negative rate of the classification. A ROC curve [38] measurement, for example the area under the curve (AUC), can also be used for choosing the best model for its overall performance. For this study, a Youden’s index [39] was used as the fitness function, considering that AUC does not measure a specific prediction accuracy which is needed in practice. More details about how to implement the GA with logistic regression can be found in references [40–42].

Separate prediction models using APACHE II, APACHE III and SAPS scores were built for comparison.

## Results

The experiments were designed to apply the GA with LR using (1) 20 HRV features only, and (2) HRV features and HRV based Euclidean distance features (40 in total). The results were then compared with the LR using APACHE II, APACHE III, SAPS or the combinations of them.

All the experimental results presented in this section were from the runs with 5 repeated fivefold cross validation, with the same dataset splits applied to all the compared models. The final sets of features selected by GA were based on 100 repeated runs. For each fivefold cross validation, the testing set included 1 non-survivor and 20% (4) of the surviving patients with 5 time points (and the following 8 time points) randomly selected for each patient (total of 5 * 5 = 25 data points). The rest of the whole dataset was used to train the models. A Youden’s index value from the fivefold cross validated testing result was used as the fitness function of GA. Table 3 shows the results from the different feature sets, with the AUC, sensitivity and specificity from the final model reported. Feature set 6 (LF_Hz, HF_Hz, LF_perc and LF/HF) were selected from the whole set of 20 original HRV features by GA. Feature sets 7 and 8 were the subset selected from the total number of 40 HRV features and HRV based DistF. We can see with the probability cut off 0.5 from the LR model, all the models produced higher specificity than the sensitivity. The models with APACHE III included as a variable/feature basically could not predict any non-survivors correctly with the default probability cut off 0.5. Overall, the HRV based features worked better than the previously adopted injury severity scores. The models created with these selected features produced higher AUC, and higher sensitivity with similar specificity. Of the previously adopted illness severity scores, only APACHE II gave a reasonably competitive result.

**Table 3:** Cross validation results from the models built with different

Set #	Features	Training			Testing		
		AUC [95% CI]	Sensitivity [95% CI]	Specificity [95% CI]	AUC [95% CI]	Sensitivity [95% CI]	Specificity [95% CI]
1	APACHE II	0.73 [0.7, 0.76]	0.23 [0.15, 0.31]	1 [1, 1]	0.74 [0.63, 0.84]	0.16 [0.01, 0.31]	1 [1, 1]
2	APACHE III	0.6 [0.56, 0.64]	0.07 [0.01, 0.13]	1 [0.99, 1]	0.59 [0.43, 0.75]	0 [0, 0]	0.98 [0.95, 1.01]
3	SAPS	0.73 [0.7, 0.75]	0.08 [0.02, 0.14]	0.96 [0.95, 0.98]	0.74 [0.65, 0.84]	0.04 [0, 0.12]	0.95 [0.91, 0.99]
4	APACHE II APACHE III	0.77 [0.74, 0.79]	0.2 [0.12, 0.28]	0.96 [0.95, 0.98]	0.65 [0.55, 0.75]	0 [0, 0]	0.96 [0.92, 1]
5	APACHE II APACHE III SAPS	0.78 [0.76, 0.81]	0.23 [0.15, 0.31]	0.96 [0.95, 0.98]	0.64 [0.53, 0.75]	0 [0, 0]	0.9 [0.84, 0.96]
6	LF_Hz HF_Hz LF_perc LFHF	0.8 [0.77, 0.83]	0.35 [0.26, 0.44]	0.97 [0.97, 0.98]	0.75 [0.66,0.84]	0.22 [0.08,0.35]	0.93 [0.89,0.97]
7	DistHR DistHF_Hz DistHF_perc DistLF/HF	0.87 [0.85, 0.9]	0.6 [0.54, 0.66]	0.96 [0.95, 0.96]	0.76 [0.64, 0.88]	0.52 [0.34, 0.7]	0.93 [0.9, 0.97]
8	HR DistHR DistHF_Hz DistVLF_perc DistHF_perc DistLF/HF	0.9 [0.87, 0.92]	0.68 [0.62, 0.73]	0.96 [0.96, 0.97]	0.77 [0.66, 0.89]	0.65 [0.48, 0.82]	0.92 [0.88, 0.96]
9	HR DistHR DistHF_Hz DistLF/HF	0.86 [0.83, 0.88]	0.62 [0.56, 0.69]	0.95 [0.94, 0.96]	0.76 [0.64, 0.88]	0.5 [0.31, 0.68]	0.93 [0.89, 0.97]

^*^Sensitivity and specificity were calculated with a probability of 0.5 from LR as a cut-off, which means when the output from LR is over than 0.5, it was classified as positive, otherwise negative

## Discussions

The novelty of this study is utilizing feature extraction strategies to predict outcome of ICU patients with only HRV parameters derived from the ECG. The Euclidean distance features extracted based on the basic HRV parameters contributed to the prediction models significantly. The limitation of this study is that there were only 5 non-survivors in the data collected, and 2 of them did not survive until the data collection was completed. With this limitation, our Euclidean distance features were calculated with eight 30-min consecutive HRV measurement. This can be expanded to a longer time period when there are more ECG records available. The proposed model used only HRV based parameters that were calculated from ECG signals to build the prediction model. As such, this methodology has the potential to be incorporated into the ICU workflow with no addition to standard patient care practices. The model produced comparable results with the earlier models that were adopted in some clinical applications. It is expected that integrating other methods for feature extraction from the ECG signals and health records may help the model achieve better prediction accuracy.

## Conclusion

The comparison between the prediction models built with different feature sets indicated that HRV based parameters alone may predict the brain injury patient outcome better than the previously adopted illness severity scores. Based on these findings, we are encouraged to test the method on a larger patient cohort and develop a practical model that is able to improve prediction of ICU brain injury patient outcome.

## Data Availability

Data are available upon request from the Menzies Health Institute Queensland for researchers who meet the criteria for access to confidential data.

## Abbreviations

APACHE: Acute Physiology and Chronic Health Evaluation
AUC: Area under the ROC
ECG: Electrocardiogram
GA: Genetic algorithm
ICU: Intensive care units
HRV: Heart rate variability
LR: Logistic regression
MODS: Multiple Organ Dysfunction Score
ROC: Receiver operator characteristic curve
SAPS: Simplified Acute Physiology Score
SOFA: Sequential Organ Failure Assessment
TBI: Traumatic brain injury
